# The mechanisms of natural products for eye disorders by targeting mitochondrial dysfunction

**DOI:** 10.3389/fphar.2024.1270073

**Published:** 2024-04-25

**Authors:** Gui-Feng Sun, Xin-Hui Qu, Li-Ping Jiang, Zhi-Ping Chen, Tao Wang, Xiao-Jian Han

**Affiliations:** ^1^ Institute of Geriatrics, Jiangxi Provincial People’s Hospital, The First Affiliated Hospital of Nanchang Medical College, Nanchang, China; ^2^ Department of Pharmacology, School of Pharmaceutical Science, Nanchang University, Nanchang, China; ^3^ The Second Department of Neurology, Jiangxi Provincial People’s Hospital, The First Affiliated Hospital of Nanchang Medical College, Nanchang, China; ^4^ Department of Critical Care Medicine, Jiangxi Provincial People’s Hospital, The First Affiliated Hospital of Nanchang Medical College, Nanchang, China

**Keywords:** eye disorder, mitochondria, mitochondrial dysfunction, natural product, oxidative stress

## Abstract

The human eye is susceptible to various disorders that affect its structure or function, including glaucoma, age-related macular degeneration (AMD) and diabetic retinopathy (DR). Mitochondrial dysfunction has been identified as a critical factor in the pathogenesis and progression of eye disorders, making it a potential therapeutic target in the clinic. Natural products have been used in traditional medicine for centuries and continue to play a significant role in modern drug development and clinical therapeutics. Recently, there has been a surge in research exploring the efficacy of natural products in treating eye disorders and their underlying physiological mechanisms. This review aims to discuss the involvement of mitochondrial dysfunction in eye disorders and summarize the recent advances in the application of natural products targeting mitochondria. In addition, we describe the future perspective and challenges in the development of mitochondria-targeting natural products.

## 1 Introduction

The human eye is responsible for detecting light and transmitting visual signals to the brain. Eye disorders encompass a range of pathological conditions that affect the function or structure of the eye. Common examples include AMD, glaucoma and DR. These disorders may stem from various factors, such as aging, genetics, inflammation and physical trauma (Miyamoto et al., 1999; Weinreb et al., 2014; Dieguez et al., 2019). It is worth noting that certain eye disorders can have multiple etiologies, and the precise cause of a specific condition may remain elusive. Nonetheless, mitochondrial dysfunction is believed to be critical in the pathogenesis and progression of most eye disorders.

Mitochondria are essential cellular organelles involved in multiple metabolic processes and are often referred as cell’s powerhouse due to their role in energy production (Han et al., 2011). Key mitochondrial functions include ATP synthesis via oxidative phosphorylation, redox regulation, calcium buffering and apoptosis control in response to extracellular and intracellular stimuli or stress. Mitochondrial quality is stringently regulated by interconnected mechanisms, such as biogenesis, dynamics and mitophagy. Given their significance in cellular homeostasis, mitochondrial dysfunctions can lead to a wide array of diseases (Lin and Beal, 2006). Numerous studies have established strong links between mitochondrial dysfunction and eye disorders, including AMD, blue light-induced damage and corneal chemical injuries (Li et al., 2018; Zhang et al., 2021; Zou et al., 2023). Insight into mitochondrial dysfunction may provide new therapeutical targets for understanding the pathophysiology of eye disorders and facilitate the development of innovative treatments.

Natural products, defined as substances or metabolites produced by living organisms, have long been used in traditional medicine and have also played pivotal role in modern drug development and clinical therapeutics. Generally considered safer than synthetic metabolites due to their reduced side effects, not all natural products have been extensively researched (Ekiert and Szopa, 2020). Recently, a growing number of studies have focused on examining the efficacy of natural products in treating eye disorders. Natural products may serve as potential therapeutic agents by targeting mitochondrial dysfunction through various signaling pathways (Guo et al., 2021; Park et al., 2021; Xu et al., 2021; Yang et al., 2022). This review aims to summarize the involvement and molecular mechanisms of mitochondrial dysfunction in eye disorders. In addition, we discuss the application of natural products in eye disorders by targeting mitochondria as well as its challenges and future perspective.

## 2 Involvement of mitochondrial dysfunction in eye disorders

### 2.1 Mitochondrial biogenesis

**FIGURE 1:**
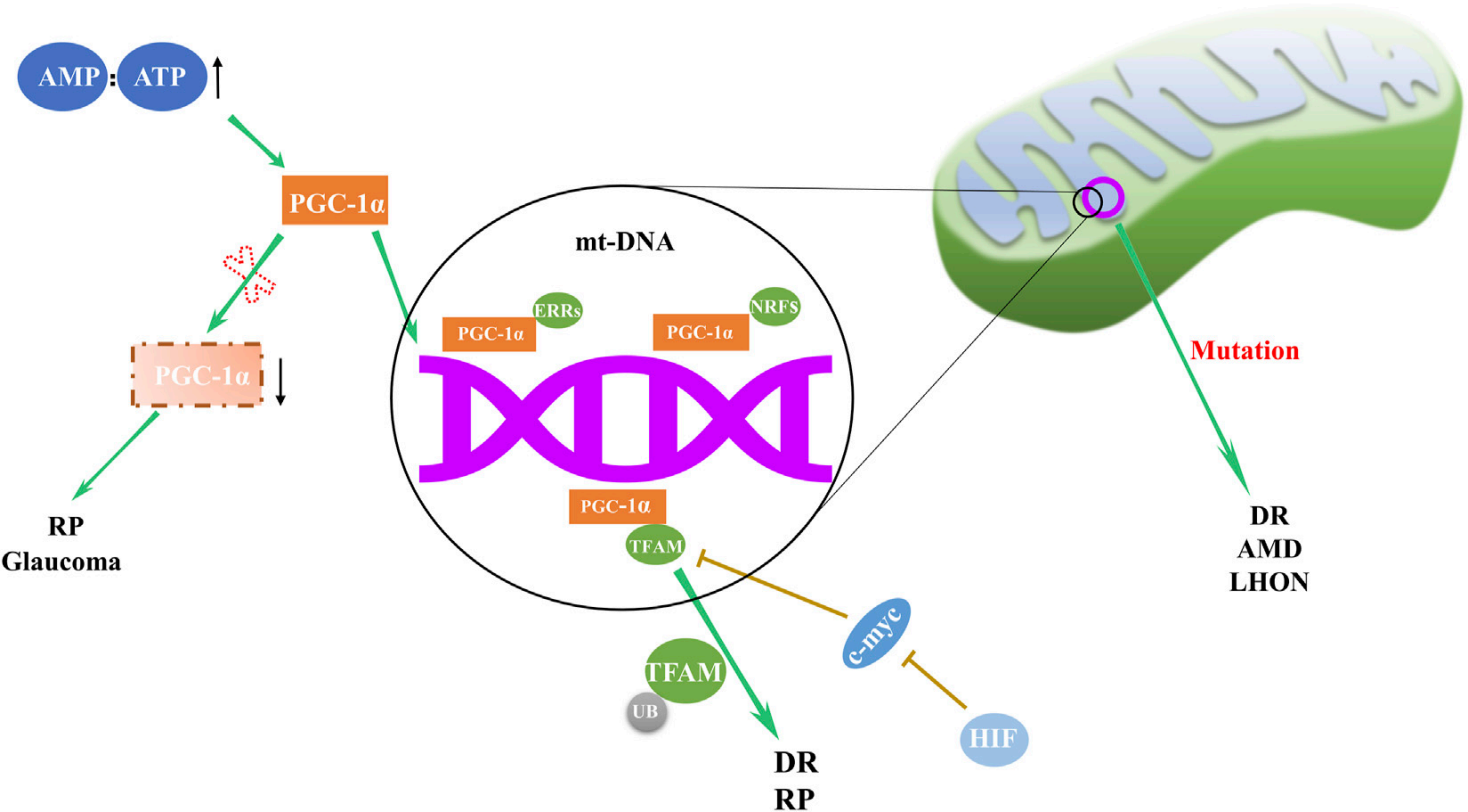
Regulation and abnormalities of mitochondrial biogenesis in ocular disorders. The orchestration of mitochondrial biogenesis involves the cooperative activity of PGC-1α and downstream transcription factors, including NRFs, ERRs, and TFAM. Deviations in this complex network are implicated in a range of eye disorders, with abnormal expression of PGC-1α and TFAM, along with mt-DNA mutations, contributing to the pathogenesis of RP, DR, AMD, LHON and glaucoma.

Mitochondrial biogenesis, the cellular mechanism responsible for augmenting mitochondrial quantity and size, is paramount to maintaining energy homeostasis. Deficiencies in mitochondria biogenesis can contribute to various eye disorders (Figure 1), such as Leber’s hereditary optic neuropathy (LHON), an affliction attributed to mt-DNA mutation (Stenton et al., 2021). Decreased expression of NRF1, TFAMB1, and TFAMA in mitochondrial biogenesis may lead to optic neuropathies (Iyer et al., 2012). In diabetic conditions, while TFAM gene transcription seems to escalate, the converse is true for its mitochondrial protein levels, which diminish, leading to subpar mitochondria copy numbers (Koh et al., 2019). Santos et al. shed light on the regulatory dynamics of TFAM, uncovering that TFAM’s ubiquitination hampers its translocation to mitochondria, thus impinging on mt-DNA transcription and impairing mitochondrial function (Santos et al., 2014). Remarkably, mitigation of TFAM ubiquitination reestablished mitochondrial homeostasis. This suggests that focusing on the post-translational modulation of TFAM may offer a novel approach to safeguard mitochondrial equilibrium and potentially alleviate the burden of DR.

In DR, retinal mitochondria become dysfunctional, and their mt-DNA is damaged (Kowluru, 2020). Mitochondrial biogenesis in the retina of patients with DR is impaired due to decreased transport of TFAM to the mitochondria (Santos et al., 2011). Modulating biogenesis through pharmaceutical or molecular approaches may offer a potential strategy to delay DR progression.

In AMD patients, retinal pigment epithelial (RPE) cells exhibit structurally and functionally defective mitochondria as well as deficient expression of Dicer enzyme (Kaneko et al., 2011; Lewis Lujan et al., 2022). Alu RNA expression is upregulated by Dicer deficiency, inducing mitochondrial membrane potential loss, reactive oxygen species (ROS) generation and release of mt-DNA into cytoplasm. This cytoplasmic mt-DNA, along with oxidative stress, activates the NLRP3 inflammasome, leading to interleukin-18 production and RPE cell apoptosis (Lewis Lujan et al., 2022).

Retinitis pigmentosa (RP), a retinal disorder rooted in mitochondrial dysfunction, manifests as progressive rod and cone cell degeneration. This process ultimately precipitates the loss of retinal light sensitivity and culminates in blindness (Pagano et al., 2021). The etiology of RP is associated with anomalies in mitochondrial biogenesis, including alterations in the regulatory factors and dynamic proteins such as PGC-1α, TFAM, Fis1, Mfn1, and Mfn2 (Ozawa et al., 2022).

In the context of neurodegenerative diseases, a common thread appears to be fluctuations in the expression of PGC-1α, master regulator of mitochondrial biogenesis (Lin et al., 2004; Leone et al., 2005; Ma et al., 2010). Significant decrease in PGC-1α expression was observed in the ganglion cell layer of inherited glaucoma model DBA/2J mice (Guo et al., 2014). This evidence underscores the pervasive role of mitochondrial dysregulation across a spectrum of retinal disorders.

### 2.2 Mitochondrial dynamics

**FIGURE 2:**
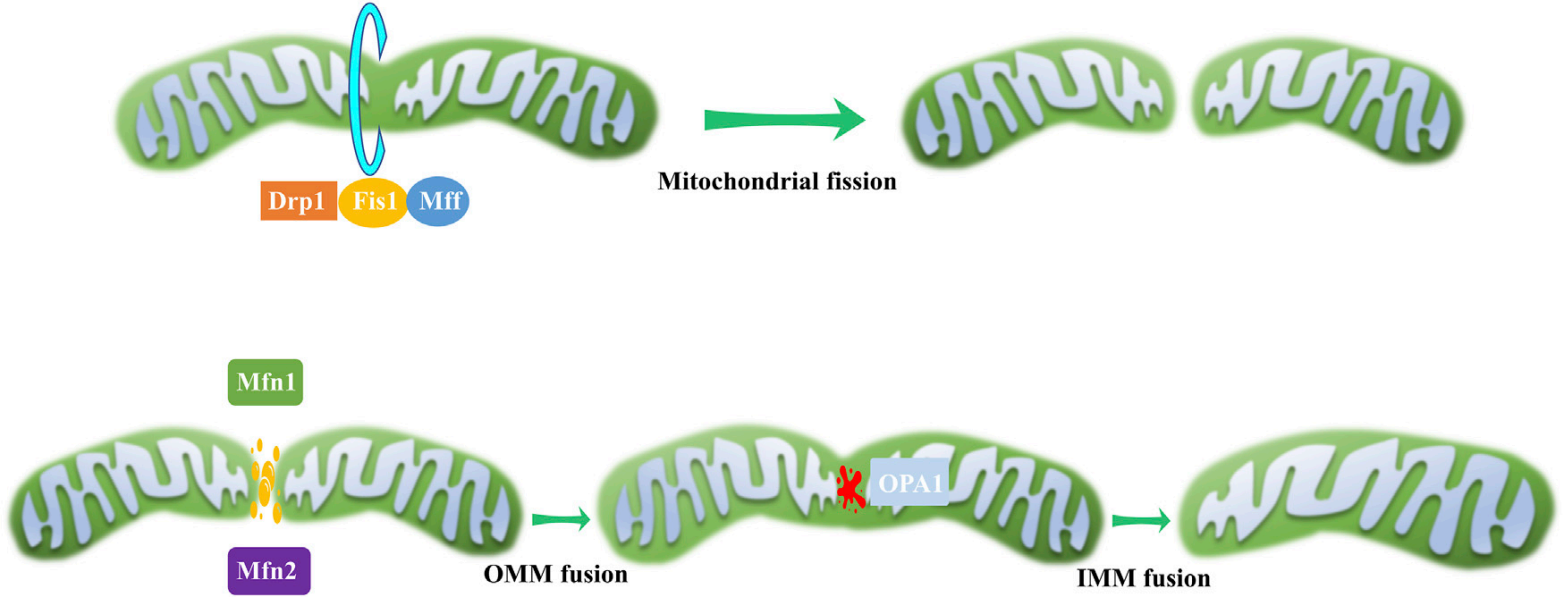
Mitochondrial dynamics. During mitochondrial fission, a scission complex composed of Drp1, Fis1, and Mff facilitates mitochondrial division. In the process of mitochondrial fusion, OMM fusion is mediated by Mfn1 and Mfn2, while IMM fusion is governed by OPA1.

Mitochondria continuously undergo fission and fusion, collectively known as mitochondrial dynamics (Figure 2), to acclimate to shifting cellular environments (Suen et al., 2008). Mitochondrial dynamics disturbance has been observed in various eye disorders (Table 1). Light-induced mitochondrial fragmentation in retina has been reported, with blue light exposure increasing Drp1 expression and decreasing Mfn2 expression (Knels et al., 2011; Marie et al., 2018). Agustina et al. reported that blue light decreased expression of OPA1 and increased expression of Drp1 in ARPE-19 cells (Alaimo et al., 2019). Dominant optic atrophy (DOA), a neuro-ophthalmic condition typified by bilateral optic nerve degeneration, is connected to mutations in OPA1 gene (Del Dotto et al., 2018). Intriguingly, heterozygous OPA1 mutations have also been linked to extra-ocular symptoms, including mitochondrial myopathy, sensorineural deafness, axonal sensory-motor polyneuropathy, chronic progressive external ophthalmoplegia, and ataxia (Amati-Bonneau et al., 2003; Li et al., 2005).

**TABLE 1:** The function of mitochondrial dynamic regulatory proteins and their abnormity in eye disorders.

Protein	Function	Abnormity	Eye disorder
Drp1	Fission	Upregulated	Light-induced retinal injury
			Chemical injury
hFis1	Forms complexes with Drp1	—	—
Mff	Forms complexes with Drp1	Mutations	Bilateral plaque like macular atrophy
			RP
OPA1	IMM fusion	Downregulated	Light-induced retinal injury
		Mutations	DOA
Mfn1	OMM fusion	Upregulated	Myopia
		Mutations	POAG
Mfn2	OMM fusion	Downregulated	Light-induced retinal injury
		Mutations	DR
			POAG

The retina’s active metabolism makes it particularly vulnerable to genetic and environmental alterations causing mitochondrial dysfunction. For example, disturbances in mitochondrial dynamics and quality control system increase susceptibility of photoreceptor and RGC to cell death, contributing to retinitis pigmentosa onset (Narayan et al., 2017; Eells, 2019; Mirra and Marfany, 2019).

Mitochondrial dynamics are also central to DR development and its associated “metabolic memory” phenomenon. Drp1 is central to maintaining mitochondrial homeostasis under these conditions and is associated with the disease’s continued progression (Mohammad and Kowluru, 2022). In diabetic milieu, retinal mitochondria exhibit swolling and damag, and Mfn2 expression decreases. Mfn2 overexpression prevents glucose-induced mitochondrial fragmentation (Duraisamy et al., 2019). Therefore, modulating Mfn2 expression and its epigenetic alterations, through molecular or pharmacological strategies, may offer potential avenues for preserving mitochondrial homeostasis and mitigating the development of DR.

In the case of corneal alkali burns, considered as a severe ophthalmic emergency and difficult to manage conservatively, Drp1-dependent mitochondrial fission has been implicated (Shi et al., 2023). It appears to mediate alkali burn-induced corneal injury by regulating inflammatory responses, oxidative stress, and corneal neovascularization (Zhang et al., 2021). These insights highlight the diverse and critical roles of mitochondrial dynamics in retinal health and various ocular disorders.

### 2.3 Mitophagy

**FIGURE 3:**
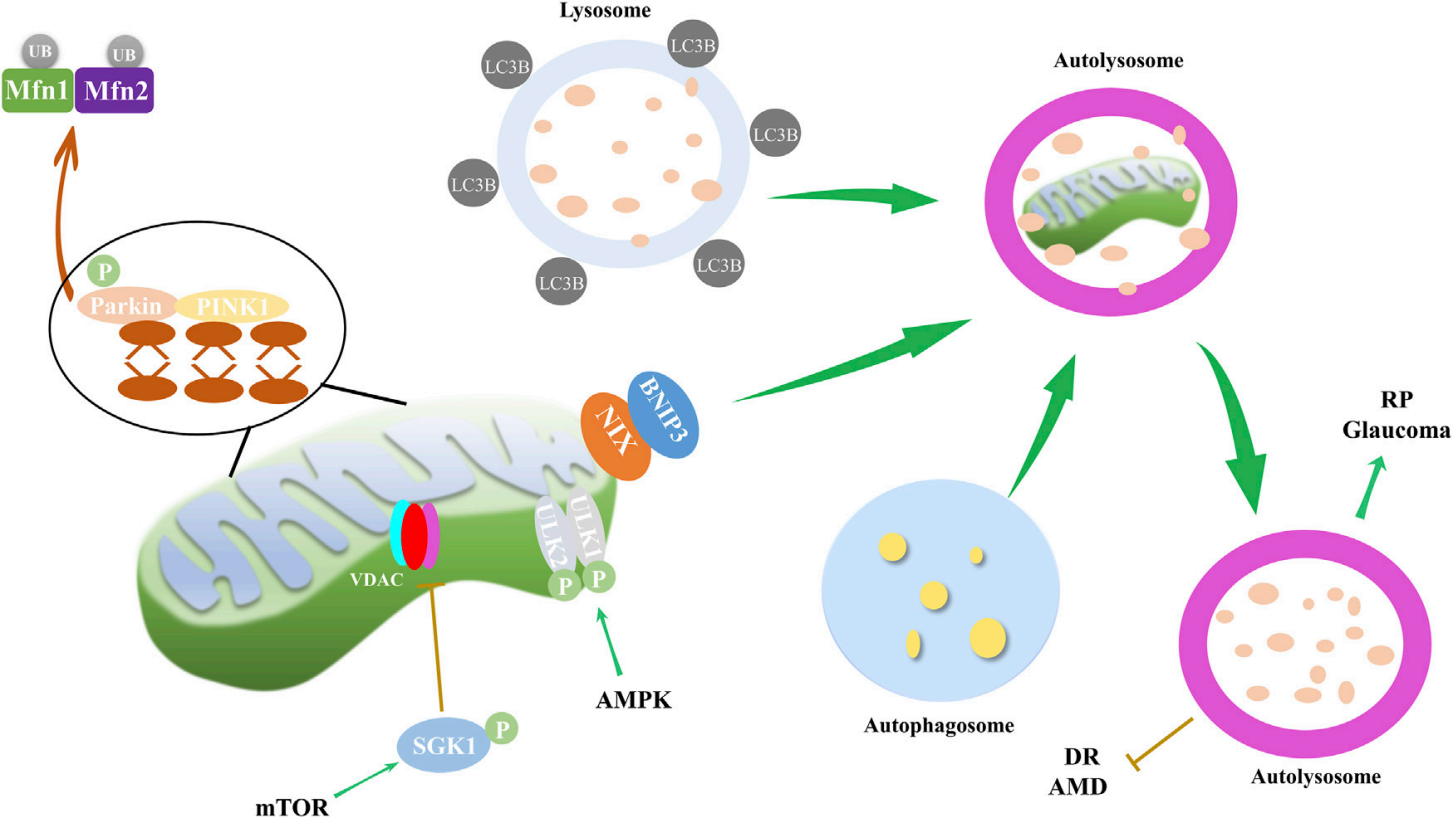
Mitophagy and its impact on ocular disorders. Mitophagy, regulated through mTOR and AMPK pathways, along with PINK1/Parkin and NIX/BNIP3 pathways, is implicated in the progression of eye disorders such as DR, RP, AMD and glaucoma. During mitophagy, PINK1 recruits and activates Parkin, and then mitochondrial Mfn1/2 are ubiquitinated by Parkin. PINK1 also recruits autophagy receptor proteins NIX and BNIP3 to mitochondria, and receptor proteins recruit LC3B. Finally, autophagosome, mitochondria and lysosome form autolysosome to dissolve and recycle damaged mitochondria. AMPK pathway mediates mitophagy through phosphorylation of ULK1/2, while mTOR inhibits VDAC by phosphorylating SGK1, thereby reducing mitophagy.

Mitophagy is a critical cellular process that involves the selective degradation of damaged or excess mitochondria through autophagy (Su et al., 2023). Proper functioning of mitochondria is essential for cellular energy homeostasis, and mitophagy prevents the accumulation of damaged organelles, which can lead to cellular damage and disease. Mitophagy has been implicated in a host of ocular disorders, as well as in neurodegenerative, metabolic, and aging-related diseases (Figure 3). The dysregulation of spliceosome-mediated mitophagy, for instance, contributes to the pathogenesis of RP (Xu et al., 2018). High glucose environments, such as those seen in DR, have been reported to inhibit cell proliferation and mitophagy via the ROS-mediated inactivation of the ROS/PINK1/Parkin signaling pathway (Zhang et al., 2019). Moreover, TXNIP and associated oxidative stress have been proposed as mechanisms for mitophagy in retinal RPE cells under sustained high glucose conditions (Singh et al., 2021). Furthermore, blue light exposure has been observed to stimulate mitophagy, as evidenced by the conversion of autophagy marker LC3B and the overexpression of mitophagy sensor PINK1 (Li et al., 2018). These findings underscore the extensive role of mitophagy in ocular health.

### 2.4 Mitochondrial redox homeostasis

**FIGURE 4:**
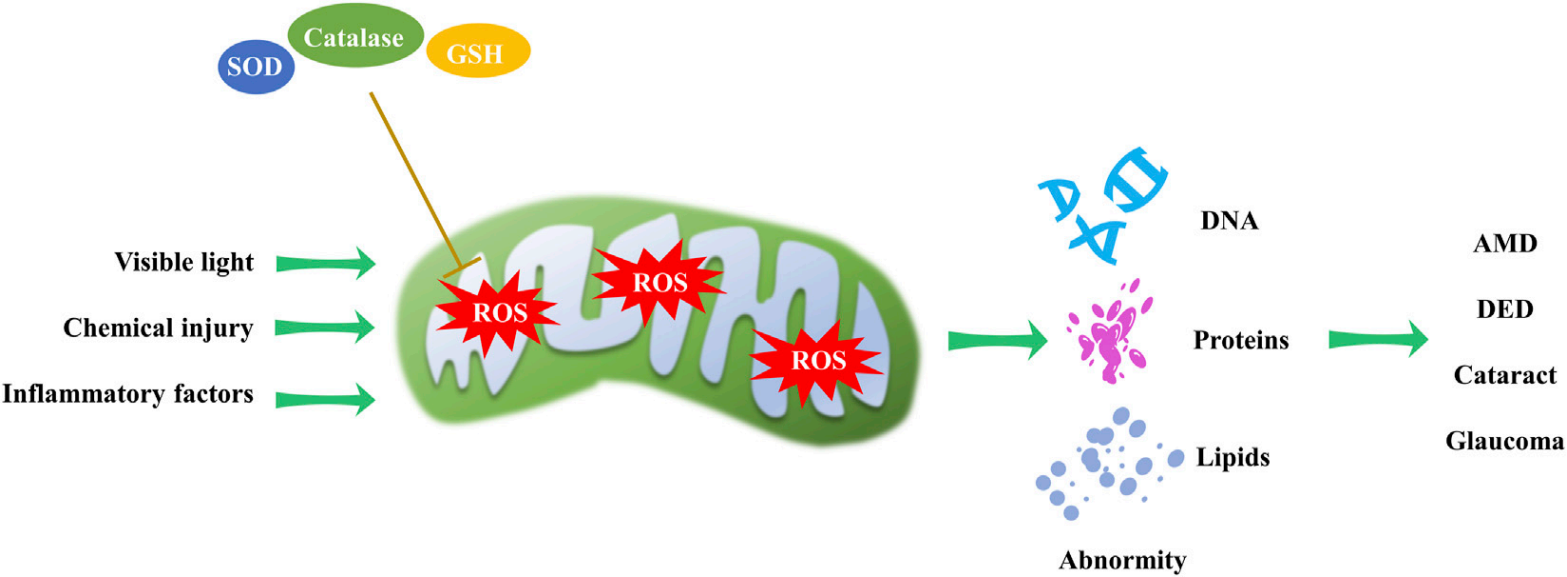
Mitochondrial redox homeostasis and its impact on eye disorders. Visible light, chemical injury and inflammatory factors induce ROS production, while antioxidants can reduce ROS levels. An imbalance in mitochondrial redox homeostasis can result in DNA, protein, and lipid abnormalities, contributing to the progression of AMD, DED, cataract and glaucoma.

Mitochondria, as the primary source of ROS in the cell, can cause damage to cellular components. Mitochondrial redox homeostasis is tightly controlled by antioxidants, including superoxide dismutases, catalases and glutathione (Seminotti et al., 2022). Dysregulation of this homeostasis can lead to oxidative stress and eye disorders (Figure 4). Various studies have shown that visible light exposure in cell cultures can trigger an overproduction of ROS, including peroxynitrite, hydroxyl free radicals, nitric oxide, hydrogen peroxide, and singlet oxygen (Godley et al., 2005; Lascaratos et al., 2007; Wood et al., 2007). This perturbation of mitochondrial redox homeostasis can subsequently lead to cataract formation (Hightower et al., 1999; Bantseev and Youn, 2006).

Intracellular redox reactions are crucial for maintaining cellular homeostasis. However, the disruption of these reactions has been implicated in the onset and progression of DR (Sharma et al., 2022). In the context of AMD, excessive ROS oxidizes macromolecules such as nucleic acids, lipids, and proteins, potentially causing structural and functional alterations. Excessive ROS and oxidized lipoproteins can trigger protein misfolding, aggregation, and chronic activation of the innate immune response (Ferrington et al., 2016; Jadeja and Martin, 2021; Tang et al., 2021).

The trabecular meshwork, the anterior chamber tissue responsible for aqueous humor drainage, is fortified with antioxidant defenses. Despite this, it remains vulnerable to mitochondrial oxidative damage that can impair its endothelial function, increase intraocular pressure, and initiate glaucoma (Nair et al., 2021). Additionally, in dry eye disease (DED), augmented oxidative stress has been strongly linked to the etiology of corneal epithelial alterations. Chronic oxidative stress exposure activates cell regulatory molecules involved in corneal surface disorders associated with dry eye conditions (Nakamura et al., 2007; Cejkova et al., 2008). These examples highlight the far-reaching implications of mitochondrial redox homeostasis in ocular diseases.

### 2.5 Apoptosis

Apoptosis, also known as programmed cell death, is an orderly and controlled process that is activated by many cell stresses, including mitochondrial damage, growth factor deprivation, disruption of the cytoskeleton, accumulation of unfolded proteins, and hypoxia. The two major pathways leading to apoptosis are extrinsic pathway and intrinsic pathway. The intrinsic pathway commences with mitochondrial outer membrane permeabilization, releasing cytochrome c into the cytoplasm and activating caspases-3 and caspases-9 (Sparrow and Cai, 2001). B-cell lymphoma 2 (Bcl-2) family proteins critically regulate mitochondrial permeability, with pro-apoptotic members (Bax and Bak) facilitating the release of pro-apoptotic factors and anti-apoptotic members (Bcl-2 and Bcl-xl) inhibiting this process (Ma et al., 2020).

**FIGURE 5:**
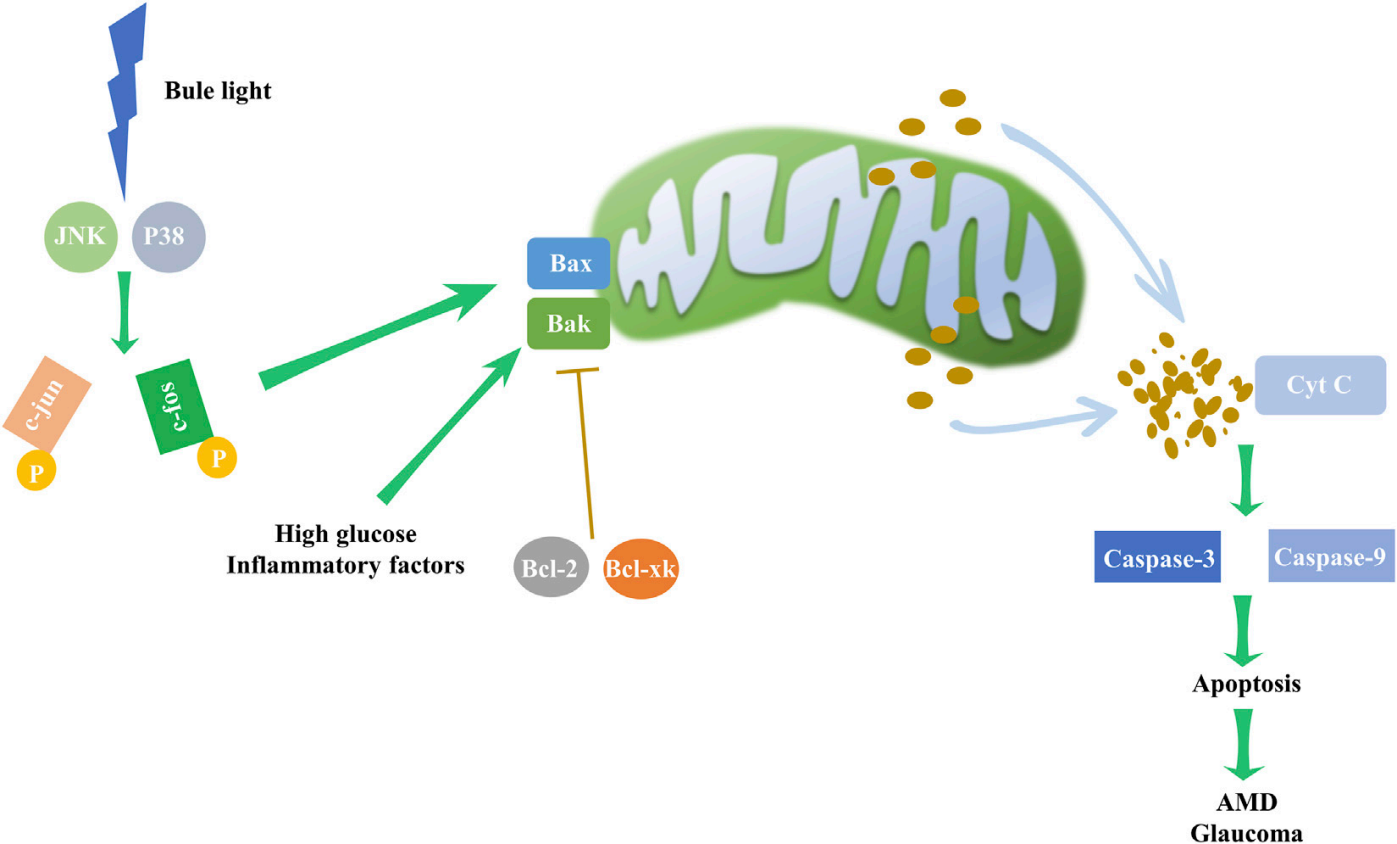
Intrinsic apoptosis pathway and its impact on eye disorders. Environmental factors, such as high glucose levels and inflammation, may upregulate the expression of Bax and Bak. In particular, blue light may elevate the levels of JNK and P38, boosting c-jun and c-fos phosphorylation, subsequently driving the expression of Bax and Bak, which are pivotal to intrinsic apoptosis pathway initiation. The pathway, however, is inhibited by the counteracting activity of Bcl-2 and Bcl-xl. Disruption of this delicate balance activates the mitochondrial apoptosis pathway, triggering cytochrome c release and escalating caspase-3 and caspase-9 activity, resulting in apoptosis. These mechanistic disruptions may underpin the development of AMD and glaucoma.

Apoptosis dysregulation contributes to a range of ocular disorders (Figure 5). For instance, blue light exposure was reported to induced apoptosis in RGC cells, with continuous activation of JNK and p38 pathways leading to c-jun and c-fos phosphorylation, which subsequently triggers apoptosis (Huang et al., 2014; Li et al., 2018). In retinal endothelial cells (RECs), several stressors can induce apoptosis, such as high glucose levels and inflammatory factors (Hu et al., 2022; Kong et al., 2022).

Glaucoma-related RGC death is primarily caused by apoptosis, which can be initiated through neurotrophin deprivation (Osborne et al., 1999; Weinreb and Khaw, 2004; Erdurmus et al., 2011). In primary open-angle glaucoma (POAG) patients, significantly reduced brain-derived neurotrophic factor (BDNF) levels adversely affect RGC and trabecular meshwork cell survival, correlating with disease severity (Ghaffariyeh et al., 2011).

Mitogen-activated protein kinase (MAPK) signaling intermediates’ alterations in expression or function contribute to AMD pathogenesis (Kyosseva, 2016). For example, increased JNK1 or its activation leads to apoptosis in a mouse model of exudative AMD (Du et al., 2013) and the high level of phosphorylated ERK may cause more Choroidal neovascularization (CNV) in neovascular AMD (Li Y. et al., 2022). These findings further underscore the integral role of apoptosis regulation in ocular diseases.

## 3 Modulation of mitochondrial function in eye disorders by natural products

The therapeutic potential of natural metabolites, particularly plant extracts, has garnered significant attention in recent years as alternative solutions for various health and wellness concerns. As shown in Table 2, these metabolites exhibit potent biological effects and have potential applications in the treatment of numerous diseases and conditions.

**TABLE 2:** Therapeutic effect of natural products in eye disorders.

Natural product	Category	Source	Function	Model	Reference
Berberine	alkaloid	*Coptis chinensis* Franch. [Ranunculaceae]	Attenuates oxidative stress Inhibits modified LDL	AMD (cell, 5–100 μM; mouse, 100–200 mg/kg) DR (cell, 5–100 μM; rat, 100–200 mg/kg)	Fu et al., 2016 Tian et al., 2013 Song et al., 2016 Zhai et al., 2020
Resveratrol	Polyphenol	*Polygonum aviculare* L. [Polygonaceae]	Attenuates oxidative stress	AMD (cell, 30–50 μM) GO ((cell, 30–50 μM)	Nashine et al., 2020 Kim et al., 2015
Carotenoids	Terpene	*Daucus carota subsp. Sativus* (Hoffm.) Schübl. and G.Martens [Apiaceae]	Attenuates oxidative stress Anti-inflammatory Reduces high intraocular pressure	AMD (cell, 1–10 μM; mouse, 1–100 mg/kg) Glaucoma (cell, 1–10 μM)	Bone et al., 1988 Bone et al., 1997 Bernstein et al., 2001 Izumi-Nagai et al., 2007 Sasaki et al., 2009 Bian et al., 2012 Mares, 2015
Magnolol	Polyphenol	*Magnolia officinalis* Rehder & E.H.Wilson [Magnoliaceae]	Attenuates oxidative stress Maintains mitochondrial membrane potential	Cataract (cell, 5–150 μM)	Lo et al., 1994 Yao et al., 2009
Eriodictyol	Polyphenol	*Styphnolobium japonicum* (L.) Schott [Fabaceae]	Anti-inflammatory	DR (rat, 1–10 mg/kg)	Bucolo et al. (2012)
Hyperoside	Polyphenol	*Hypericum monogynum* L. [Hypericaceae]	Attenuates oxidative stress Anti-inflammatory Prevents apoptosis	DR (cell, 5–80 μM; rat, 20–100 mg/kg)	Sun et al., 2021 Wu et al., 2020
Kaempferol	Polyphenol	*Enicostema axillare subsp. littorale* (Blume) A.Raynal [Gentianaceae]	Attenuates oxidative stress Prevents apoptosis Inhibits angiogenesis	DR (cell, 10–100 μM)	Xu et al., 2017 Du et al., 2018, Al Sabaani, 2020
Polyphenol-enriched cocoa	Polyphenol	*Cocos nucifera* L. [Arecaceae]	Attenuates oxidative stress	DR (cell, 0.1–10 μg/mL; rat, 24–190 mg/kg)	Duarte et al. (2015)
Baicalein	Polyphenol	*Goodyera schlechtendaliana* Rchb.f. [Orchidaceae]	Attenuates oxidative stress Anti-inflammatory Prevents apoptosis	DR (cell, 20–100 μM; rat, 75–150 mg/kg) Retinal ischemia injury (cell, 0.05–0.5 nmol; rat, 75–150 mg/kg)	Pan et al., 2021 Liu et al., 2010 Chao et al., 2013 Yang et al., 2009
Acacetin	Polyphenol	*Turnera diffusa* Willd. ex Schult. [Passifloraceae]	Attenuates oxidative stress Anti-inflammatory Protects nerve	DED (cell, 0.001–3 μM; mouse, 5–45 mg/kg)	Singh et al., 2020 Xiao et al., 2019 Wu et al., 2021 Xie et al., 2022
		*Saussurea involucrata* (Kar. & Kir.) Sch.Bip. [Asteraceae]			
(−)-Epigallocatechin gallate	Polyphenol	*Citrus × aurantium f. deliciosa* (Ten.) M.Hiroe [Rutaceae]	Attenuates oxidative stress Increases visual function	DR (cell, 10–50 μM; rat, 25–50 mg/kg) Retinal ischemia injury (cell, 10–50 μM; rat, 25–50 mg/kg) RP (rat, 25–50 mg/kg)	Fernando and Soysa, 2016 Silva et al., 2013 Perdices et al., 2022
Xanthohumol	Polyphenol	*Humulus lupulus* L. [Cannabaceae]	Attenuates oxidative stress	DED (cell, 0.1–100 μM; mouse, 1–16.9 mg/kg)	Liu et al., 2015 Yao et al., 2015 Ghosh et al., 2021
Polydatin	Polyphenol	*Reynoutria japonica* Houtt. [Polygonaceae]	Attenuates oxidative stress Mitigates orbital muscle adipose tissue expansion Decreases lipid droplet accumulation Anti-inflammatory	DED (cell, 0.1–88 μM; rat, 50 mg/kg) GO (cell, 0.1–88 μM; rat, 50 mg/kg)	Ravagnan et al., 2013 Li et al., 2020 Park et al., 2019
Gastrodin	Phenol	*Gastrodia elata* Blume. [Orchidaceae]	Attenuates oxidative stress Anti-inflammatory Prevents apoptosis	Glaucoma (cell, 10–100 μM)	Yuan et al., 2019 Liu et al., 2021 Wang et al., 2017 Li et al., 2022

For AMD and DR, berberine (BBR) derived from *Coptis chinensis* Franch. [Ranunculaceae] has shown therapeutic promise. It inhibits modified low-density lipoprotein (LDL)-induced Müller cell injury by activating the AMPK signaling pathway (Fu et al., 2016). Moreover, BBR mitigates leukocyte-mediated vascular endothelial damage, decreases antioxidant enzyme activities, and combats DR and AMD involving oxidative stress (Tian et al., 2013; Song et al., 2016). BBR’s capability to inhibit oxidative stress and cell apoptosis via NF-*κ*B signaling pathway deactivation highlights its therapeutic potential for DR (Zhai et al., 2020). Resveratrol, a plant polyphenol, is noted for reducing intracellular ROS levels and enhancing mitochondrial quality in an AMD model (Nashine et al., 2020), and attenuating oxidative stress in Graves’ orbitopathy (GO) (Kim et al., 2015).

### 3.1 AMD, cataract and glaucoma

Carotenoids, namely, lutein (L), zeaxanthin (Z), and meso-zeaxanthin (meso-Z), are known to confer retinal protection (Bone et al., 1988; Bone et al., 1997; Bernstein et al., 2001). Ubiquitously found in plants, L and Z have been shown to mitigate oxidative stress and inflammation, thus playing a crucial role in preventing AMD (Izumi-Nagai et al., 2007; Sasaki et al., 2009; Bian et al., 2012). Significantly, these are the only two carotenoids documented to offer protection against lens opacities and cataract formation, attributable to their antioxidative properties (Mares, 2015). Additionally, L and Z may also exert protective effects on the trabecular meshwork, thereby decreasing the risk of high intraocular pressure (IOP) and subsequent glaucoma (Bernstein et al., 2001).

Magnolol, a metabolite isolated from the Chinese botanical drug *Magnolia officinalis* Rehder & E.H.Wilson [Magnoliaceae], has received increasing attention due to its antioxidant activity (Lo et al., 1994). Magnolol has been shown to inhibit ROS generation, prevent loss of mitochondrial membrane potential, and curtail cytochrome c release from mitochondria in H_2_O_2_-treated HLE B-3 cells. (Yao et al., 2009). By thwarting oxidative stress, magnolol implies a promising therapeutic strategy for cataract prevention.

Eriodictyol demonstrates potent anti-inflammatory properties, attenuating plasma lipid peroxidation and preserving the integrity of the blood-retinal barrier, thereby safeguarding retinal health (Bucolo et al., 2012). Hyperoside (Hyp), a plant-derived flavonoid, possesses multifaceted properties, including anti-cancer, anti-inflammatory, and anti-oxidative effects (Sun et al., 2021). It exhibits a protective role in diabetes-induced retinopathy, as evidenced in diabetic rat models, through the mitigation of oxidative stress, cell damage inhibition, and apoptosis prevention (Wu et al., 2020). Polyphenol-enriched cocoa offers retinal protection by enhancing the SIRT1 pathway in streptozotocin-induced diabetic rats, safeguarding the retina from oxidative stress damage (Duarte et al., 2015).

Kaempferol, a beneficial flavonoid in retinal protection, impedes angiogenesis in human retinal endothelial cells. This effect is mediated by downregulating the Src-Akt1-Erk1/2 signaling pathway and the placental growth factor, and vascular endothelial growth factor (VEGF) (Xu et al., 2017). Moreover, it bolsters cell survival, shields RPE cells from H_2_O_2_-induced oxidative damage and apoptosis by suppressing ROS generation, downregulating VEGF, and upregulating superoxide dismutase (Du et al., 2018). The protective influence of kaempferol against H_2_O_2_-induced ARPE-19 damage is attributed to its antioxidant and anti-inflammatory attributes, facilitated partly through the stimulation of nuclear accumulation, activation, and deacetylase ability of SIRT1, while concurrently inhibiting PARP1 (Al Sabaani, 2020).

### 3.2 DR and retinal ischemia injury

Baicalein, an active metabolite extracted from botanical drugs, has therapeutic potential due to its antioxidative and anti-inflammatory properties (Pan et al., 2021). Liu et al., 2010 highlighted the antioxidative capabilities of baicalein in the context of retinal ischemia. Moreover, the pre-treatment of baicalein has shown efficacy in modulating apoptotic factors, including Bax and Bcl-2, thus attenuating retinal cell apoptosis in a rat retinal ischemia/reperfusion model (Chao et al., 2013). Furthermore, baicalein, when administered orally, safeguards retinal vessels and neurons from DR-induced dysfunction and apoptosis. This protective effect is attributed to its ability to curb retinal inflammatory processes governed by microglia and Müller cells and to attenuate the release of pro-inflammatory cytokines such as IL-18, TNF-α, and IL-1β (Yang et al., 2009).

(−)-Epigallocatechin gallate (EGCG), the most prevalent catechin-based flavonoid in green tea leaves, has demonstrated substantial potential as a retinal protective agent (Fernando and Soysa, 2016). EGCG confers protection against ischemia injury (Peng et al., 2008) and DR (Silva et al., 2013). Its protective scope also extends to RP, where it not only lessens the visual function loss in P23H rats but also elevates the levels of antioxidant enzymes and reduces oxidative damage (Perdices et al., 2022).

Acacetin, a naturally occurring flavone found in various plants, including *Turnera diffusa* Willd. ex Schult. [Passifloraceae] and *Saussurea involucrata* (Kar. & Kir.) Sch.Bip. [Asteraceae], shows a gamut of pharmacological and biochemical activities (Singh et al., 2020). These encompass antioxidant, anti-inflammatory, and neuroprotective effects (Xiao et al., 2019; Wu et al., 2021). Importantly, Acacetin has demonstrated the ability to inhibit inflammatory responses by augmenting NLRP3 ubiquitination, thereby suggesting potential therapeutic benefits for depression-associated DED (Xie et al., 2022).

Xanthohumol, a naturally occurring prenylated chalconoid derived from *Humulus lupulus* L. [Cannabaceae], is a known promoter of the transcription of phase II antioxidant enzymes. It achieves this through the facilitation of the dissociation of Kelch-like ECH-associated protein 1 (Keap1) from nuclear factor erythroid 2-related factor 2 (NRF2) (Liu et al., 2015). Additionally, the chalconoid structure of Xanthohumol confers direct ROS scavenging activity, thereby broadening its therapeutic potential (Yao et al., 2015). As a result, Xanthohumol has exhibited cytoprotective effects in human corneal epithelial cells and a mouse desiccating stress/scopolamine model, further validating its prospective role in ocular therapeutics (Ghosh et al., 2021).

Polydatin (resveratrol-3-O-β-mono-D-glucoside, PD), a resveratrol glycoside found in Reynoutria japonica Houtt. [Polygonaceae], exhibits notable effects on orbital muscle adipose tissue expansion and lipid droplet accumulation. (Ravagnan et al., 2013). It employs a NRF2-mediated oxidative stress response involving the Keap1/NRF2/ARE pathway (Li et al., 2020). Furthermore, PD impedes hyperosmolar stress-induced inflammation by attenuating NF-κB translocation to the nucleus and diminishing the expression of pro-inflammatory markers such as TNF-α, IL-6, IL-1β, and MMP9. Importantly, PD also inhibits the hyperosmolar stress-induced NLRP3 inflammasome pathway and ROS production, suggesting its promising therapeutic potential for DED and GO (Park et al., 2019).

On the other hand, Gastrodin, an active metabolite of the traditional Chinese botanical drug *Gastrodia elata* Blume. [Orchidaceae], possesses anti-inflammatory, antioxidative, and anti-apoptotic properties (Yuan et al., 2019; Liu et al., 2021). This points to its potential role in treating retinal neurodegenerative diseases marked by retinal ganglion cell death (Wang et al., 2017). Notably, Gastrodin can protect retinal ganglion cells from hypoxia/reoxygenation injury by activating the PI3K/AKT/NRF2 pathway, thus offering potential avenues for glaucoma therapy (Li S. et al., 2022).

The increasing application of natural products in ophthalmology signifies their untapped pharmacological potential. However, hurdles such as under targeting, low bioavailability, subpar pharmacological activity, high metabolic decomposition rates, and uncertain pharmacological mechanisms persist. These challenges impede the translation of plant-derived natural products from basic research to clinical practice (Mahran et al., 2017; Rao et al., 2019; Takke and Shende, 2019). Hence, further research is paramount to enhance structural modifications and develop novel pharmaceutical agents based on natural products (Gunasekaran et al., 2014; Gaston et al., 2020).

## 4 Conclusion

As cellular powerhouses, mitochondria are vital for an array of cellular activities, producing the energy necessary for these processes. A growing body of research suggests a close association between mitochondrial dysfunction and common ocular disorders, including glaucoma, AMD and DR. Encouragingly, certain treatment modalities are transitioning from experimental stages to clinical applications. Particularly, the utilization of natural metabolites shows considerable promise, as numerous studies have demonstrated their potential efficacy in treating diverse ocular disorders. However, it is important to note that further investigations are necessary to comprehensively elucidate the underlying mechanisms of their biological effects. Additionally, a critical aspect that warrants attention is the safety assessments of these natural metabolites in the context of ocular disorders. Addressing these aspects will not only enhance our understanding of their therapeutic potential but also contribute to their safe and effective translation into medical practices. In light of the accumulating evidence, it is evident that natural metabolites could assume a significant role in the future of medical and personal care domains.

## Figures and Tables

**Table:** 

**AMD**	Age-related macular degeneration
**DR**	Diabetic retinopathy
**ATP**	Adenosine triphosphate
**PGC-1α**	Peroxisome proliferator-activated receptor-γ coactivator
**ERRs**	Estrogen-related receptors
**NRFs**	Nuclear respiratory factors
**AMPK**	AMP-activated protein kinase
**AMP**	Adenosine monophosphate
**TFAM**	Mitochondrial transcription factor A
**mt-DNA**	Mitochondrial deoxyribonucleic acid
**HIF**	Hypoxia-inducible factor
**c-myc**	Cellular-myelocytomatosis viral oncogene
**LHON**	Leber’s hereditary optic neuropathy
**NRF1**	Nuclear respiratory factor 1
**RPE**	Retinal pigment epithelial
**RNA**	Ribonucleic acid
**ROS**	Reactive oxygen species
**NLRP3**	Nod-like receptor thermal protein domain associated protein 3
**RP**	Retinitis pigmentosa
**Fis1**	Mitochondrial fission protein 1
**Mfn1**	Mitofusin 1
**Mfn2**	Mitofusin 2
**Drp1**	Dynamin-related protein 1
**Mff**	mitochondrial fission factor
**OPA1**	Optic atrophy 1
**OMM**	Outer mitochondrial member
**IMM**	Inner mitochondrial member
**Oma1**	Overlapping with the m-AAA protease 1 homolog
**ARPE-19**	Human retinal pigment epithelial cell
**DOA**	Dominant optic atrophy
**RGC**	Retinal ganglion cell
**mTOR**	Mammalian target of rapamycin
**PINK1**	PTEN induced putative kinase 1
**BNIP3**	Bcl-2 interacting protein 3
**NIX**	Nip-like protein X
**mt-ROS**	Mitochondrial reactive oxygen species
**DNA**	Deoxyribonucleic acid
**IL**	Interleukin
**TXNIP**	Thioredoxin interacting protein
**LC3B**	Microtubule-associated protein 1 light chain 3 beta
**DED**	Dry eye disease
**Bcl-2**	B-cell lymphoma 2
**Bax**	B-cell lymphoma 2 associated x protein
**Bcl-xl**	B-cell lymphoma-extra large
**JNK**	Jun N-terminal kinase
**REC**	Retinal endothelial cell
**POAG**	Primary open-angle glaucoma
**BDNF**	Brain-derived neurotrophic factor
**MAPK**	Mitogen-activated protein kinase
**ERK**	Extracellular signal-regulated Kinase
**CNV**	Choroidal neovascularization
**BBR**	Berberine
**LDL**	Low-density lipoprotein
**NF-κB**	Nuclear factor kappa-B
**IOP**	Intraocular pressure
**Hyp**	Hyperoside
**SIRT1**	Sirtuin 1
**VEGF**	Vascular endothelial growth factor
**PARP1**	Poly (ADP-ribose) polymerase 1
**TNF-α**	Tumor necrosis factor alpha
**EGCG**	(−)-Epigallocatechin gallate
**Keap1**	Kelch-like ECH-associated protein 1
**NRF2**	Nuclear factor erythroid 2-related factor 2
**PD**	Polydatin
**ARE**	Antioxidant response element
**MMP9**	Matrix metalloproteinase 9
**PI3K**	Phosphoinositide 3-kinase
**GO**	Graves’ orbitopathy
